# Cathepsin H indirectly regulates morphogenetic protein-4 (BMP-4) in various human cell lines

**DOI:** 10.2478/v10019-011-0034-3

**Published:** 2011-10-08

**Authors:** Matija Rojnik, Zala Jevnikar, Bojana Mirkovic, Damjan Janes, Nace Zidar, Danijel Kikelj, Janko Kos

**Affiliations:** 1 Department of Pharmaceutical Biology, Faculty of Pharmacy, University of Ljubljana, Ljubljana, Slovenia; 2 Department of Pharmaceutical Chemistry, Faculty of Pharmacy, University of Ljubljana, Ljubljana, Slovenia

**Keywords:** bone morphogenetic protein 4, cancer, cathepsin H, human cell lines, proteolytic enzymes

## Abstract

**Background:**

Cathepsin H is a cysteine protease considered to play a major role in tumor progression, however, its precise function in tumorigenesis is unclear. Cathepsin H was recently proposed to be involved in processing of bone morphogenetic protein 4 (BMP-4) in mice. In order to clarify whether cathepsin H also regulates BMP-4 in humans, its impact on BMP-4 expression, processing and degradation was investigated in prostate cancer (PC-3), osteosarcoma (HOS) and pro-monocytic (U937) human cell lines.

**Materials and methods:**

BMP-4 expression was founded to be regulated by cathepsin H using PCR array technology and confirmed by real time PCR. Immunoassays including Western blot and confocal microscopy were used to evaluate the influence of cathepsin H on BMP-4 processing.

**Results:**

In contrast to HOS, the expression of BMP-4 mRNA in U937 and PC3 cells was significantly decreased by cathepsin H. The different regulation of BMP-4 synthesis could be associated with the absence of the mature 28 kDa cathepsin H form in HOS cells, where only the intermediate 30 kDa form was observed. No co-localization of BMP-4 and cathepsin H was observed in human cell lines and the multistep processing of BMP-4 was not altered in the presence of specific cathepsin H inhibitor. Isolated cathepsin H does not cleave mature recombinant BMP-4, neither with its amino- nor its endopeptidase activity.

**Conclusions:**

Our results exclude direct proteolytic processing of BMP-4 by cathepsin H, however, they provide support for its involvement in the regulation of BMP-4 expression.

## Introduction

Cathepsin H (CTSH) (EC.4.22.16), a cysteine protease, is ubiquitous in cells and tissues, but its physiological role is poorly understood.^1^,^2^ CTSH acts mainly as an aminopeptidase but also exhibits limited endopeptidase activity.^3^ In addition to heavy and light chains, which are typical of a number of mammalian papain-like cysteine proteases, mature CTSH also contains an octapeptide EPQNCSAT, termed the mini-chain, that originates from the propeptide and is bound to the mature form by a disulphide bond.^4^ The mini-chain is essential for the aminopeptidase activity of CTSH.^5^

CTSH is synthesized as a preproenzyme of 41 kDa which is proteolytically activated through a multistep process to a 30 kDa intermediate form and finally to the single chain mature form of 28 kDa.^6^ This form can be further processed to a 22 kDa heavy chain and a 5–6 kDa light chain.^2^,^3^,^7^

CTSH was identified to play an important role in the establishment and development of a functional tumor vasculature and increases the metastatic potential of human hepatoma cell lines.^8^–^10^ Expression of CTSH differs in breast carcinoma^11^, colorectal cancer^12^, melanoma^13^, head and neck carcinoma^14^,^15^, glioma^16^ and prostate cancer^17^ and from that in normal tissue. CTSH is associated with physiological and pathological processes of the lung.^18^–^20^ Furthermore, it is involved in the N-terminal processing step of surfactant protein C in type II pneumocytes and pro-granzyme B in cytotoxic lymphocytes.^21^,^22^ Bone morphogenetic protein 4 (BMP-4) is a potential target for CTSH endopeptidase activity during the differentiation of mouse lungs, and lower activity was proposed to lead to marked accumulation of BMP-4 protein and disruption of branching morphogenesis.^23^

BMP-4, as a member of the transforming growth factor β family is involved in the development of many organs and tissues and was shown to play a role in cancer progression.^24^–^26^ BMP-4 is synthesized as a large inactive precursor which is proteo-lytically cleaved to the mature protein in a multistep process.^27^ Non-processed BMP-4 is targeted to the lysosomes for degradation which can lead to severe loss of BMP-4 activity in specific tissues.^28^,^29^

Lü *et al*.^23^ presented evidence that CTSH and BMP-4 expression coincides during branching morphogenesis in mouse models. They showed that inhibition of CTSH leads to accumulation of the mature BMP-4 in embryonic mouse lungs. However, they failed to demonstrate the cleavage of BMP-4 by mature 28 kDa CTSH *in vitro*.

In this study we have evaluated the role of human CTSH in BMP-4 processing and regulation at the mRNA and protein level, using various human cell lines.

## Materials and methods

### Cell culture

Human U937 pro-monocytic (CRL-1593.2; ATCC, Manassas, VA, USA) and WEHI231 mouse lymphoma cells (CRL-1702 cell line; ATCC, Manassas, VA, USA) were maintained in advanced RPMI 1640 (Gibco, Invitrogen, Scotland) supplemented with 2mM glutamine (Sigma, St. Louis; MO, USA), antibiotics (Penicillin-Streptomycin, Sigma, St. Louis, USA) and 10% fetal bovine serum (FBS) (HyClone, Logan, USA). U937 cells were differentiated with PMA (50 nM) (Sigma, St. Louis; MO, USA) for 24 h to achieve attachment. Human osteosarcoma HOS cells (CRL-1543 cell line, ATCC, Manassas, VA, USA) were grown in minimal essential medium (MEM) (Sigma, St. Louis, MO, USA) supplemented with 2.2 g/L sodium bicarbonate (Riedel de Haën, St. Louis, MO, USA), 2 mM glutamine, antibiotics and 10% FBS. Human prostate cancer PC-3 cells (cell line CRL-1435, ATCC, Manassas, VA) were cultured in DMEM/F12 (1:1) medium (Gibco, Invitrogen, Scotland) supplemented with antibiotics, 2 mM glutamine and 10% FBS.

### Western blot analysis

Lysates of HOS, PC-3 and PMA differentiated U937 cells were prepared in 50 μl of 0.05 M sodium acetate buffer (pH 6) with added 1mM EDTA, 0.1 M NaCl, 0.25% Triton X-100 (lysis buffer). Complete lysis of the cells was achieved by three 5 to 7 s sonication cycles. Clear supernatants were obtained after centrifugation at 4°C and 16200 g for 15 min. Total protein concentration was determined by the Bradford method using Coomassie Plus Protein Assay reagent (Pierce, Thermo Fischer Scientific) with BSA (Sigma, St. Louis; MO, USA) as standard. Samples containing 100 μg of proteins were heated at 100°C in reducing sample buffer for 10 min, separated by 12% SDS-PAGE and transferred to nitrocellulose membranes. The molecular weight of the proteins was determined using SeeBlue® Plus2 Pre-Stained Standard (Invitrogen, USA). The membrane was blocked in 5% skimmed milk in Tween-PBS for 30 min and incubated with sheep polyclonal anti-cathepsin H (5 μg/ml)^30^ or goat polyclonal anti-BMP4 (dilution 1:400, sc-6896, Santa Cruz Biotechnology, CA, USA) antibodies overnight at 4°C. After washing with Tween-PBS the membrane was incubated with secondary HRP conjugated rabbit anti-sheep (1:10000, sc-2770, Santa Cruz Biotechnology, CA, USA) or donkey anti-goat (1:10000, Santa Cruz Biotechnology, CA, USA) antibodies for 2 h at room temperature.

### Real Time PCR analysis

PCR-arrays (Common Cytokine PCR Array; PAHS-021, SABiosciences, MD, USA) were used according to the manufacturer’s protocol. RNA was isolated from U937 cells treated with 0.5 μM native human liver CTSH (nCTSH) and compared to RNA from control cells. Data was analyzed using RT² Profiler PCR Array Data Analysis (SABiosciences, MD, USA). Quantitative Real Time PCR (qPCR) was performed as reported.^31^ Total RNA was isolated from U937, HOS and PC-3 cells using RNeasy Mini kit (Qiagen, Hilden, Germany) according to manufacturer’s protocol. For cDNA synthesis 1 μg of total mRNA was reverse transcribed using OmniscriptRT Kit (Qiagen, Hilden, Germany). qPCR was carried out on an ABI PRISM 7000 apparatus (Applied Biosystems, Life Technologies Corporation, CA, USA) in a total reaction volume of 25 μl containing 5 μl cDNA of different concentrations, BMP4 QuantiTect Primer Assay (Qiagen, Hilden, Germany) and Maxima™ SYBR Green/ROX qPCR Master Mix (2x) (Fermentas International Inc, Ontario, Canada). The cycling program was 2 min at 50°C, 10 min at 95°C, followed by 40 cycles (15 s at 95°C and 60 s at 60°C). Multiple housekeeping genes were checked (the primer sequences were found in the Real Time PCR Primer and Probe Data Base) for their stability using geNorm normalization. The data was normalized to the endogenous controls HPRT and GAPD for U937, HPRT and YWHAZ for HOS and PC-3. A melting curve of PCR products (60–95°C) was also performed to ensure the absence of artefacts. All assays were performed in parallel and in three biological repetitions.

### Confocal immunofluorescence microscopy

HOS and PC-3 cells were grown on glass coverslides in 24-well plates for 24 h prior to the experiment; U937 cells were differentiated with PMA (50 nM) for 24 h. WEHI231 were seeded on slides and cytospinned for 6 min at 2500 g. Before labeling, cells were fixed with 4% paraformaldehyde in PBS (pH 7.4) for 30 min and permeabilized with 0.1% Triton X-100 in PBS (pH 7.4) for 10 min. Non-specific staining was blocked with 3% BSA in PBS (pH 7.4). CTSH was labeled with primary mouse monoclonal anti-CTSH 1D10 antibody (10 μg/ml of 3%BSA in PBS).^30^ Goat polyclonal anti-human BMP4-N16 antibody was used for BMP-4 labeling (Santa Cruz Biotechnology, CA, USA). After 2 h of incubation, cells were washed three times with PBS and treated with Alexa 488-labeled rabbit anti-mouse and Alexa 555-labeled donkey anti-goat (2:1000, Molecular Probes, Invitrogen, USA) antibodies for 2 h. After washing with PBS, ProLong Antifade kit (Molecular Probes, Invitrogen, USA) was mounted on dried coverslides and allowed to dry overnight at 4°C. Cells were studied by fluorescence microscopy at room temperature using a Carl Zeiss LSM 510 confocal microscope (Carl Zeiss Inc., Jena, Germany); immersion oil was used as imaging medium. Images were analyzed using Carl Zeiss LSM image software 3.0.

### Synthesis of specific synthetic irreversible inhibitor of CTSH - H_2_N-Ser(OBzl)-CHN_2_ (CTSHi)

(S)-(9H-Fluoren-9-yl)methyl (1-(benzyloxy)-4-dia-zo-3-oxobutan-2-yl)carbamate (A). Triethylamine (0.175 mL, 1.258 mmol) in THF (3 mL) was added to a stirred solution of Fmoc-L-Ser(Bzl)-OH (1, 500 mg, 1.198 mmol) in THF (6 mL) at −20°C under argon, followed by the addition of ethyl chloroformate (0.120 mL, 1.258 mmol) in THF (3 mL). The mixture was stirred for 30 minutes at −5°C, after which the precipitated Et_3_NH^+^Cl^−^ was filtered off. Acetonitrile (5 mL) and trimethylsilyldiazomethane (2.0 M sol. in hexane, 1.198 mL, 2.395 mmol) were added to the filtrate and the mixture was stirred overnight at +4°C. Ethyl acetate (50 mL) was added and organic phase washed successively with 10% aq. citric acid (2 × 20 mL), sat. aq. NaHCO_3_ (2 × 20 mL) and brine (2 × 20 mL). The organic phase was dried over Na_2_SO_4_, filtered and the solvent evaporated under reduced pressure. The crude product was purified with flash column chromatography using ethyl acetate/petroleum ether (1:4) as eluent to afford 2 as a pale yellow solid (378 mg, 0.856 mmol). Yield: 71%; Rf = 0.43 (EtOAc/petroleum ether = 1:1); IR (KBr): ν = 3552, 3414, 3311, 3076, 2859, 2106 (C=N=N), 1800, 1696, 1630, 1534, 1450, 1385, 1293, 1266, 1103, 1029, 736 cm-1. 1H NMR (DMSO-d6): δ 3.60-3.69 (m, 2H, CH_2_), 4.21-4.37 (m, 4H, CH, CH, CH_2_), 4.49 (s, 2H, CH_2_Ph), 6.07 (s, 1H, CHN_2_), 7.26-7.44 (m, 9H, Ar-H), 7.74 (d, 2H, J = 7.2 Hz, Ar-H), 7.84 (d, 1H, J = 8.1 Hz, NH), 7.90 (d, 2H, J = 7.5 Hz, Ar-H). MS (ESI): m/z (%) = 464 ([M+Na]^+^, 33), 414 ([MH–N_2_]^+^, 35). (S)-3- Amino-4-(benzyloxy)-1-diazobutan-2-one (B). To a solution of A (150 mg, 0.340 mmol) in acetonitrile (10 mL), diethylamine (10 mL) was added and the mixture stirred at room temperature. After 20 min the mixture was concentrated under reduced pressure and purified with flash column chromatography using dichloromethane/methanol (20:1) as eluent, to afford 3 as an yellow oil (20 mg, 0.091 mmol). Yield: 27%; Rf = 0.23 (CH_2_Cl_2_/MeOH = 10:1); 1H NMR (CDCl_3_): δ 1.73 (br s, 2H, NH_2_), 3.59-3.70 (m, 3H, CH, CH_2_), 4.55 (s, 2H, CH_2_Ph), 5.79 (s, 1H, CHN_2_), 7.31-7.40 (m, 5H, Ph).

Analytical TLC was performed on silica gel Merck 60 F254 plates (0.25 mm), using visualization with UV light and ninhydrin. Column chromatography was carried out on silica gel 60 (particle size 240–400 mesh). 1H NMR spectra were recorded at 300 MHz on a Bruker AVANCE DPX300 spectrometer in CDCl3 or DMSO-d6 solution, with TMS as the internal standard. IR spectra were recorded on a Perkin-Elmer 1600 FT-IR spectrometer. Mass spectra were obtained using a VGAnalytical Autospec Q mass spectrometer.

Inhibition constants for CTSH (k_2_ 2938.3 s^−1^M^−1^) and CTSB (k_2_ 5.1 s^−1^M^−1^) were similar as referred in the literature.^32^

### Cleavage of mature human recombinant BMP-4 protein

nCTSH was tested for its ability to degrade human recombinant BMP-4 protein (GenwayBio, San Diego, CA, USA). BMP-4 (7.5 μM) was incubated with nCTSH (0.75 μM) for 1.5 h at 37°C in CTSH activity buffer (pH 6.8) using the same protocol as Obermajer *et al*.^33^ nCTSH was pretreated with 10 μM CTSHi or DMSO for controls. Samples were analyzed using 12% SDS-PAGE followed by Western blot or reverse-phase HPLC (Shimadzu Coorporation, Japan) using a Discovery BIO Wide Pore C5 column (Sigma, St.Louis, MO, USA) with UV-VIS detector.

### Statistical analysis

SPSS PC software (Release 13.0) was used for statistical analysis. Statistical significance was evaluated by Student’s t test. P values of less than 0.05 were considered to be statistically significant.

## Results

### Different processing forms of CTSH

**FIGURE 1:**
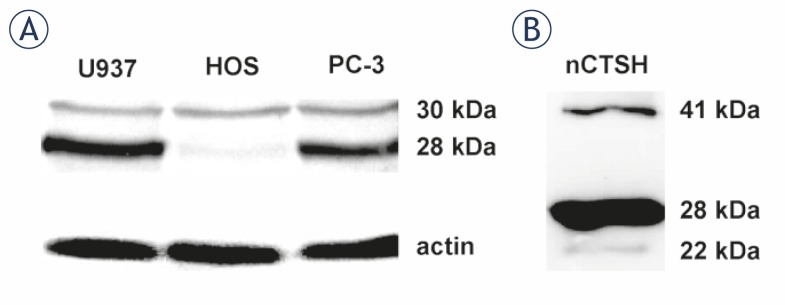
CTSH processing forms. (A) Different forms of CTSH were detected in human cell lines and (B) in the sample of nCTSH using Western blot. CTSH was visualized with sheep anti-CTSH primary pAb and rabbit anti-sheep horse radish peroxidise (HRP) labeled secondary antibody. The intermediate 30 kDa CTSH form is present in all of the selected cell lines, while the mature 28 kDa CTSH form is missing in HOS cells. A procathepsin H (41 kDa) and single chain form (22 kDa) could be detected in smaller amounts compared to the mature forms. nCTSH contains the procathepsin H, the mature and heavy chain forms.

The presence of different processing forms of CTSH was determined by Western blot in U937, HOS and PC-3 cell lines (Figure 1A). A 30 kDa intermediate form was detected in all selected cell lines, while the mature single chain 28 kDa form was absent in HOS cells. Only small quantities of the 22 kDa heavy chain (from the two-chain form) were detected in human cell lines (data not shown). The sample of CTSH isolated from human liver (nCTSH)^30^ contains predominantly mature 28 kDa form (Figure 1B), with a small amount of 22 kDa heavy chain. It is likely that the 30 kDa form was further processed to mature and heavy chain forms.

### CTSH regulates the expression of BMP family genes

**FIGURE 2:**
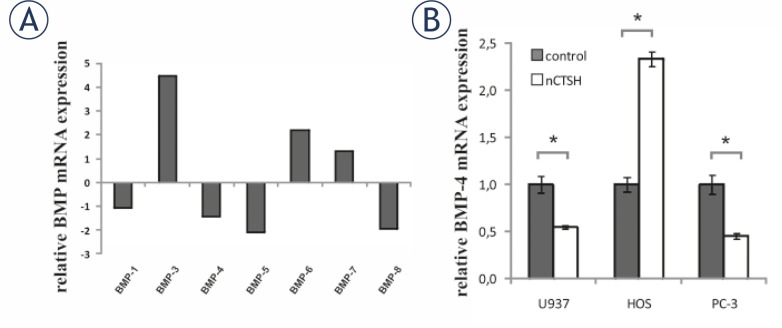
CTSH dependent cytokine mRNA expression. (A) The effect of CTSH on the mRNA expression of cytokines from BMP family. Differentiated U937 cells were incubated with 0.5 μM nCTSH and the mRNA levels were screened by PCR-arrays enabling simultaneous screen of the of 84 cytokine genes. The expression of BMP-2 is not presented, while it was probably an artifact, as shown by the analysis of the melting curve. (B) The influence of CTSH on BMP-4 mRNA expression was further evaluated with quantitative real time PCR analysis in U937, HOS and PC-3 cell lines. Cells were treated with 0.5 μM nCTSH for 24 h. The mRNA levels obtained from control samples were normalized to 1. Each bar represents the mean±SD. Ns, non-significant, *P<0.05.

The effect of CTSH on human cytokine mRNA levels was screened by PCR-array, enabling simultaneous expression of 84 cytokine genes. Incubation of differentiated U937 cells with nCTSH (0.5 μM) induced significant changes in the expression of several BMP family genes. BMP-3, 6 and 7 were significantly up-regulated, while BMP-4, 5 and 8 were down-regulated (Figure 2A). The expression of BMP-1 was not altered. CTSH dependent regulation of BMP-4 mRNA expression in human cell lines was further confirmed by specific quantitative real time PCR analysis (Figure 2B). After the addition of nCTSH, BMP4 mRNA levels in HOS increased 2.33 ± 0.08 fold, while BMP-4 mRNA levels in U937 and PC-3 were decreased 0.55 ± 0.01 and 0.45 ± 0.03 fold.

### CTSH is not involved in the proteolytic processing of BMP-4

**FIGURE 3:**
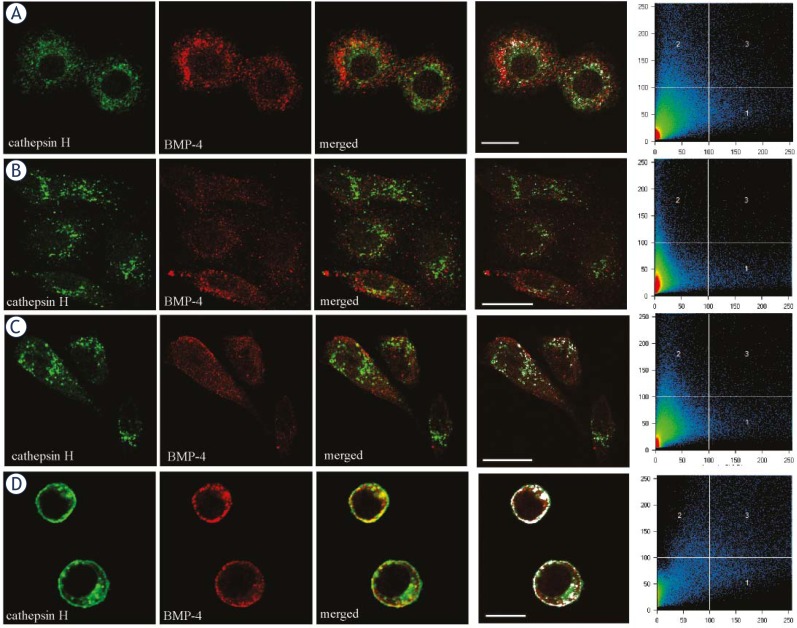
Co-localization of CTSH (green fluorescence) and BMP-4 (red fluorescence) in U937 (A), HOS (B), PC-3 (C) and WEHI231 (D) cells. Weak or no co-localization was found in selected human cells (A, B, C), while clear co-localization can be seen in mouse WEHI231 cell line (D). CTSH was labeled with 1D10 monoclonal primary antibody and anti-mouse AlexaFluor™488 secondary antibody. BMP-4 was labeled with primary goat polyclonal anti-BMP-4 antibody (Santa Cruz) and anti-goat AlexaFluor™ 555 secondary antibody. The sites of co-localization are shown in white (frame 4) and correspond to the pixels that are over the threshold in both channels (frame 5). Scale bars represent 5 μm (A and D) and 20 μm (B and C).

**FIGURE 4:**
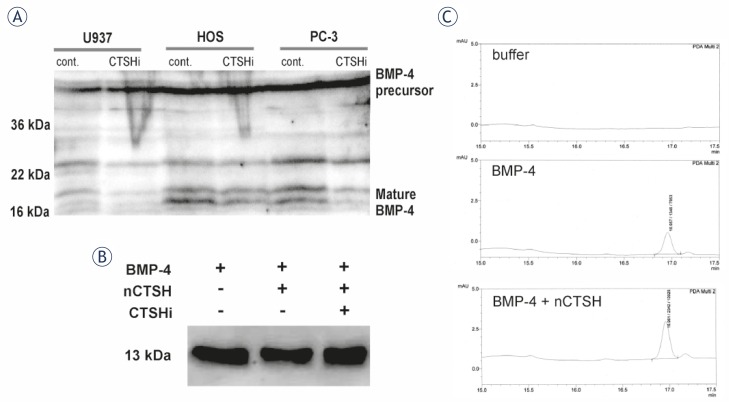
CTSH processing of mature BMP-4 protein. (A) Human cell lines were treated with 5 μM CTSHi for 24 h and then cell lysates were analyzed using Western blot. Proteins (50 μg) from cell lysates were separated on SDS-PAGE and transferred on to PVDF membrane. BMP-4 was detected with anti-BMP-4 N16 antibody (Santa Cruz) and then with secondary antibody labelled with HRP. The molecular mass in kDa is indicated on the left hand side of the blots. Molecular mass of the mature BMP-4 is detected to be approximately 18 kDa. (B) Using Western blot we analyzed the products of the reaction between mature human recombinant BMP-4 and nCTSH. Mature human recombinant BMP-4 was incubated for 1.5 h at 37ºC in CTSH activity buffer (lane 1), with 60 ng of nCTSH in CTSH activity buffer (lane 2) and 60 ng of nCTSH in CTSH activity buffer that was pre-treated for 10 min with 10 μM CTSHi (lane 3). Mature human recombinant BMP-4 has a molecular mass of 13 kDa. (C) Using reverse phase HPLC we analyzed CTSH activity buffer (buffer), mature human recombinant BMP-4 in CTSH activity buffer (BMP-4) and the products of the reaction between mature human recombinant BMP-4 and nCTSH in CTSH activity buffer (BMP-4 + nCTSH). BMP-4 was eluted in the fraction around 17.0 min.

No significant co-localization of CTSH and BMP-4 was observed in human U937, HOS, PC-3 cells under confocal microscopy. To present the difference between the co-localization of CTSH and BMP-4 in human and in mouse cells mouse WEHI231 cells were used revealing strong co-localization of both proteins (Figure 3). Furthermore, the role of CTSH in the intracellular degradation of mature BMP-4 was determined using a specific synthetic irreversible inhibitor of CTSH – H_2_N-Ser(OBzl)-CHN_2_ (CTSHi; 5 μM).^32^ The inhibition of CTSH was followed by SDS-PAGE and Western blot analysis (Figure 4A). No increase of mature BMP-4 was observed in U937, HOS and PC-3 cells treated with CTSHi, indicating that CTSH inhibition does not have a direct impact on BMP-4 processing or degradation in human cells. Intramolecular cleavage of BMP-4 by CTSH endopeptidase activity was excluded *in vitro* by incubation of recombinant BMP-4 and nCTSH. No additional protein bands appeared on Western blots (Figure 4B). The products of the proteolytic cleavage were also analyzed by reverse phase HPLC to detect possible N-terminal cleavage of BMP-4 by CTSH aminopeptidase activity. No changes in the height or area of the BMP-4 peak were observed after incubation with nCTSH, excluding significant amounts of aminopeptidase processing (Figure 4C).

## Discussion

BMP-4 regulates cell proliferation, differentiation, apoptosis and cell fate throughout mammalian development.^34^ CTSH has been suggested to regulate its recycling or degradation in the developing lung of mice.^23^ In the current study, we identified CTSH as a regulator of BMP-4 mRNA expression in human cell lines, however, we excluded direct pro-teolytic processing of BMP-4 by CTSH amino- or endopeptidase activity.

CTSH expression is ubiquitous, with very high levels in the kidney.^35^ There is growing evidence that its expression changes under various pathological conditions, the most extensively studied being its role in cancer.^17^,^36^,^37^ However, its natural substrates and mechanism of action are not known. Using Western blot we have detected different processing forms of CTSH in human prostate cancer (PC-3), osteosarcoma (HOS) and pro-monocytic (U937) cell lines. Whereas the 41 kDa proenzyme and 30 kDa intermediate were present in all cell lines, the mature 28 kDa form was missing in human osteoblasts HOS. The 28 kDa single chain is believed to be the most important form of active CTSH in exerting specific aminopeptidase activity.^5^ However, del Re *et al*.^36^ demonstrated that in colorectal carcinoma the expression of the 30 kDa form is decreased, while the expression of the mature 28 kDa form is increased in tumor comparing to normal tissue, thus showing the importance of different CTSH processing forms.

We attempted to identify potential targets of CTSH proteolytic activity in human cells, using PCR array technology. In U937 cells a strong association between CTSH and mRNA expression of BMP family members was observed. The results of quantitative real time PCR demonstrate that CTSH affects BMP-4 mRNA expression differently in selected cell lines. In U937 and PC-3 cells the addition of nCTSH decreased the expression of BMP-4, whereas in HOS cells the level of BMP-4 mRNA was increased. Interestingly, in HOS cells, where the 28 kDa single chain form of CTSH is missing, the trend of BMP-4 mRNA regulation by nCTSH is opposite to that in U937 and PC-3 cells, where this form is present. This implies that CTSH dependent regulation of BMP-4 mRNA expression is probably controlled by the mature chain form of CTSH, possibly interfering with the promoter of BMP-4 biosynthesis.

Proteolytic processing by BMP-4 is dependent on the proteolytic activity at the two different sites, S1 and S2, in BMP-4 pro-domain.^38^ Cleavage of the S2 site is enhanced in slightly acidic conditions, as occurs in subcellular organelles like endosomes and lysosomes. Other studies also stressed the need of lysosomal (and proteasomal) function for processing of BMP-4.^28^ Therefore, CTSH as a lysosomal protease could be involved in BMP-4 proteolytic cleavage and, indeed, the endopeptidase activity of the 22 kDa form of CTSH was proposed to cleave BMP-4.^23^ Confocal microscopy was used to determine whether CTSH protein is co-localized with BMP-4, thus being capable of its proteolytic degradation. No co-localization between the two proteins was found in human cell lines, indicating that CTSH and BMP-4 are not present in the same subcellular organelles, so it is unlikely that CTSH is involved directly in the processing or degradation of BMP-4. Furthermore, inhibition of CTSH did not alter the processing of BMP-4 or increase the levels of mature BMP-4 in human cells. These results strongly suggest that CTSH has no direct role in intracellular BMP-4 proteolytic cleavage in the selected human cells. On the other hand, in mouse WEHI231 cells, the significant co-localization of CTSH and BMP-4 indicates the possible involvement of CTSH in BMP-4 protein processing and showing presumably different processing of BMP-4 in mice compared to humans

To confirm a possible indirect action of nCTSH on human recombinant BMP-4 by either endopeptidase or aminopeptidase activity *in vitro*, Western blot analysis was performed, clearly showing that nCTSH, which contains both the mature 28 kDa and heavy chain 22 kDa, does not cleave mature BMP-4 as an endopeptidase. Moreover, using reverse phase HPLC analysis of BMP-4 following incubation with nCTSH, we demonstrated that the N-terminal of human recombinant BMP-4 is also not cleaved by its aminopeptidase activity. The latter is consistent with the fact that CTSH is not able to hydrolyze substrates by its aminopeptidase activity if proline is at the S_1_′ position^39^, as is the case at the N-terminal of mature human BMP-4, which starts with the Ser-Pro-Lys-His-His- sequence.^40^

BMP-4 plays an important role in the differentiation and proliferation of neural^41^, and colorectal cancer stem cells^42^ and is a critical component in regulating hematopoietic stem cell function.^43^ The involvement of cysteine cathepsins in the migratory potential and differentiation of stem cells was studied before.^44^ Our results imply that CTSH might be important in the processes of stem cell differentiation by regulating the expression of BMP-4.

In conclusion, we have demonstrated that CTSH activity is not directly involved in proteolytic processing of BMP-4 in human cells but can regulate mRNA expression of BMP family members, depending on the presence of different processing forms of CTSH. However, the mechanisms of regulation of its mRNA expression, as well as the impact of CTSH on members of BMP family other than BMP-4, remain to be elucidated.
